# Effort versus Reward: Preparing Samples for Fungal Community Characterization in High-Throughput Sequencing Surveys of Soils

**DOI:** 10.1371/journal.pone.0127234

**Published:** 2015-05-14

**Authors:** Zewei Song, Dan Schlatter, Peter Kennedy, Linda L. Kinkel, H. Corby Kistler, Nhu Nguyen, Scott T. Bates

**Affiliations:** 1 Department of Plant Pathology, University of Minnesota, Saint Paul, MN 55108, United States of America; 2 Department of Plant Biology, University of Minnesota, Saint Paul, MN 55108, United States of America; 3 USDA ARS Cereal Disease Laboratory, Saint Paul, MN 55108, United States of America; Graz University of Technology (TU Graz), AUSTRIA

## Abstract

Next generation fungal amplicon sequencing is being used with increasing frequency to study fungal diversity in various ecosystems; however, the influence of sample preparation on the characterization of fungal community is poorly understood. We investigated the effects of four procedural modifications to library preparation for high-throughput sequencing (HTS). The following treatments were considered: 1) the amount of soil used in DNA extraction, 2) the inclusion of additional steps (freeze/thaw cycles, sonication, or hot water bath incubation) in the extraction procedure, 3) the amount of DNA template used in PCR, and 4) the effect of sample pooling, either physically or computationally. Soils from two different ecosystems in Minnesota, USA, one prairie and one forest site, were used to assess the generality of our results. The first three treatments did not significantly influence observed fungal OTU richness or community structure at either site. Physical pooling captured more OTU richness compared to individual samples, but total OTU richness at each site was highest when individual samples were computationally combined. We conclude that standard extraction kit protocols are well optimized for fungal HTS surveys, but because sample pooling can significantly influence OTU richness estimates, it is important to carefully consider the study aims when planning sampling procedures.

## Introduction

Terrestrial ecosystems are inhabited by an astonishing diversity of soil microorganisms [1], which play critical roles in global geochemical cycles and ecosystem functioning [2,3]. A large portion of this diversity was essentially hidden from microbial ecologists until DNA-based techniques eliminated the constraints of culture-dependent community surveys [4]. Since then, the technology for sequencing DNA has advanced at a staggering rate, with progress currently allowing for the generation of millions of microbial community sequences from environmental samples in very short periods of time [5].

Soil fungi are an important component of terrestrial microbial diversity due to their considerable influence on aboveground biodiversity and primary productivity, symbiotic relationships with most land plants, and as drivers of the global soil carbon cycle [3,6–8]. The number of publications using high-throughput sequencing (HTS) to characterize fungal communities in soil is growing exponentially (S1 Fig), and these studies reveal a high degree of diversity and endemism as well as functional redundancy among these fungi [9–11]. Although general outlines for the fungal community HTS workflow have been published [12], few studies have examined the protocol used in preparing samples for HTS, with some notable exceptions [13–15]. Such considerations are important, as standardization of HTS procedures will allow for cross-study comparisons of microbial HTS datasets, which will ultimately benefit many emerging frontiers in microbial ecology [16].

Modification of sample preparation procedures has been previously shown to influence fungal community characterization in culture-independent non-HTS studies. For example, increasing the amount of soil used in DNA extraction [17–19] or the use of sample pooling methods [20] can enhance capture of rare species. Additional steps in extraction protocol have also been shown to increase DNA yield [21–23] as well as the number of species recovered [24–26]. Conversely, species richness gains can also be achieved through regulating DNA template amount to improve PCR efficiency [12,27]. The influence of these different procedural modifications on our ability to characterize the richness of soil fungal communities, however, has not been sufficiently examined for the deep sequencing levels now possible using HTS.

To assess the role of modified procedures on species richness capture and address the need for standardization, we examined sample preparation protocols used in HTS surveys of soil fungi. Samples were collected at two sites in the Midwestern United States representing prairie and forest soils. Specifically, we examined the effect of extracting DNA from different volumes of soil (10 g, 1 g, 0.25 g) and using various extraction protocol modifications (freeze/thaw, sonication, 65°C water bath) on recovered fungal richness. Along with these treatments, we also examined fungal richness outcomes using varying PCR DNA template amounts (10 ng, 20 ng, 40 ng) and compared different sample pooling methods (physical vs. computational) to assess their ability to capture fungal community richness and composition at both sites.

## Methods

Sample collection in Cedar Creek grant through Cedar Creek Ecosystem Science Reserve of the University of Minnesota. Sample collection in Cloquet forest grant through Cloquet Forest Center of the University of Minnesota.

### Soil sampling

In fall 2013, soils were collected from two sites: the University of Minnesota Cedar Creek Ecosystem Science Reserve (CCR, 45°24'13" N, 93°11'20" W) and the University of Minnesota Cloquet Forestry Center (CFC, 46°40'45" N, 92°31'08" W). These sites represent distinct biome types, prairie and forest, respectively. CCR has mean monthly temperature range from -10°C to 22°C with an annual mean temperature at 6.7°C and an annual precipitation of 800 mm. The soil is highly sandy (93%) of the Zimmerman series [28]. CFC has mean monthly temperatures range from -14°C to 19°C with an annual mean temperature at 4°C and an annual precipitation of 761 mm. The soil is a loamy sand of the Omega and Cloquet series [29]. At CCR, soils were collected from the base of *Andropogon gerardii* plants growing in monoculture (n = 2 samples) and polyculture (n = 2 samples). For each sample, three 10 cm deep × 2.5 cm wide cores were taken from the base of an individual *A*. *gerardii* plant and combined in the field. At CFC, soils were collected under four replicate plots containing six tree species (*Pinus strobus*, *Larix laricina*, *Picea glauca*, *Quercus rubra*, *Betula papyifera*, and *Acer saccharum*). For each sample, four 10 cm deep × 2.5 cm wide cores were taken one meter apart and combined in the field. At each site, four individual plot sample replicates were gathered. All soil samples were transported in coolers to the lab and stored at -20°C until sieving at 4 mm size to remove fine roots and any part of small woody debris prior to DNA extraction.

### Experimental design and DNA extraction

We designed our experiments to examine the effects of soil volume, DNA extraction modifications, PCR DNA template amount, and soil sample pooling on observed fungal richness and community composition. To assess soil volume, DNA was extracted from 10 g, 1 g, and 0.25 g of soil from each plot (12 samples sequenced per site, 24 samples sequenced in total). The PowerMax Soil DNA kit (MO BIO Laboratories, Carlsbad, CA, USA) was used for 10 g extractions. For the 1 g extractions, the PowerSoil kit (MO BIO Laboratories, Carlsbad, CA, USA) was used to extract DNA from four replicate 0.25 g portions of the same plot soil sample (0.25 g is the standard quantity recommended for this kit by the manufacturer). DNA concentration for each of the four replicate extractions were then quantified using a BioTek Synergy H1 plate reader (BioTek, Winoosku, VT, USA) and mixed at equal amount to prepare a single ‘1 g’ sample. An additional 0.25 g portion of the same plot soil sample was used in the PowerSoil kit to represent the kit ‘standard’ extraction. All extractions were carried out according to the manufacturer’s instruction. Homogenizing was done, as specified in the kit protocol, by vortexing 10 min on the MO BIO vortex adapters (MO BIO Laboratories, Carlsbad, CA, USA, 13000-V1-24 for PowerSoil kit, and 13000-V1-50 for PowerMax Soil kit).

In the DNA extraction kit modification experiment, 0.25 g samples of soil were added to the PowerSoil ‘bead-beater’ extraction tubes containing the kit buffer. Samples were then subjected to three different modification procedures, freeze/thaw, sonication, or heating via water bath incubation (12 treatment samples sequenced per site, 24 treatment samples sequenced in total). The freeze/thaw treatment consisted of three rounds of freezing and thawing by submersing tubes in liquid N_2_ for 5–10 seconds followed by an incubation step in a 65°C water bath for 10 min. The sonication treatment consisted of sonicating tubes for 10 min in a Branson 8200 sonicator (Thomas Scientific, Swedesboro, NJ, USA). Finally, the heating treatment consisted of incubating tubes in a 65°C water bath for 10 min. After each modification, DNA extraction proceeded according to the kit protocol instructions. The standard 0.25 g soil extraction from the soil volume experiment above served as an unmodified control for comparison.

To assess the effects of soil sample pooling and DNA template amount, four individual plot samples were combined in equal amounts (3 g from each sample) and thoroughly mixed to form a ‘bulked’ sample (hereafter referred to as the ‘physical’ pool) for each site. From these physical pool samples, DNA was extracted from 10 g, 1 g, and 0.25 g soil as describe above. DNA extracted from each of these three different soil volumes was then used as template in individual PCR reactions containing different template DNA amounts (10 ng, 20 ng, and 40 ng). Aliquots were prepared from a standardized 10 ng uL^-1^ stock concentration and added to the PCR reaction at 1 μL, 2 μL, and 4 μL volumes to achieve the desired quantity of DNA template (9 samples sequenced per site, 18 samples sequenced in total). Along with all of the aforementioned samples, for each extraction kit that was used (PowerSoil and PowerMax Soil), a soil-free extraction was conducted using the manufacturer’s protocol to serve as negative controls. These negative controls allowed us to identify contaminants introduced during the extraction process and remove them from the final analyses. Finally, a ‘mock’ fungal community consisting of DNA extracted from 25 known fungal taxa was also included in the sequencing run to allow for internal optimization of the OTU clustering method [14].

### Fungal ITS amplification and Illumina sequencing

PCR amplification of the ITS1 region of the nuclear ribosomal RNA gene was conducted using the general fungal primers ITS1-F and ITS2 adapted for Illumina sequencing [13]. Using other primer combinations mays improve amplification of certain fungal groups [30]; however, testing differences among primer pairs was not the aim of this study. Each 20 μL PCR reaction consisted of 10 μL 2x Roche FastStart PCR Master (Roche, Indianapolis, IN, USA), 0.35 μL of each forward and reverse primers (10 μM), 1.2 μL MgCl_2_ (25 mM), and 2 μL template DNA (20 ng, except in the DNA template amount experiment where 10 ng and 40 ng were also used, see above). Triplicate PCR reactions were performed for each sample using three separate annealing temperatures (50°C, 53°C, and 55°C)[14]. Thermocycling conditions included an initial denaturation step of 95°C for 10 min, 30 cycles of 95°C for 30s, annealing at 50°C, 53°C, or 55°C for 20 s, and extension at 72°C for 30 s, and a single final extension at 72°C for 8 minutes. Triplicate PCR reactions at each annealing temperature were pooled, purified using the Agincourt AMPure XP PCR purification kit (Beckman Coulter Inc., Brea, CA, USA). Purified PCR products were quantified using the PicoGreen dsDNA assay (Life Technologies, Grand Island, NY, USA) following the manufacturer’s instructions, and 30 ng of DNA from each sample was combined into a single pool for Illumina MiSeq sequencing at the University of Minnesota Biomedical Genomics Center.

### Data processing

Low quality ends of reads (< q20), primer sites, and adapter sites were trimmed from forward MiSeq Illumina reads (match error rate of e = 0.2) using Cutadapt [31]. Trimmomatic [32] was then used to trim from both ends (quality threshold set at 20) and filter out reads less than 125 bp long. Sequences with ambiguous bases and homopolymers greater than 9 bp were removed in Mothur [33]. All sequences files were then combined into a single fasta file with valid QIIME labels [34]. To generate a *de novo* database of operational taxonomic units (OTUs), sequences were dereplicated and singletons were removed in USEARCH [35]. Sequences were then clustered at 97% similarity using the UPARSE algorithm implemented in USEARCH, which included chimera detection and filtering. Subsequently, to reduce the number of spurious OTUs, a second round of clustering was conducted at 95% similarity using the UCLUST algorithm in QIIME, as Nguyen *et al*. (2014) found that ‘chaining’ OTU clustering algorithms generated more accurate OTU bins in a similarly processed dataset [14]. The representative OTU list after the ‘chaining’ clustering were filtered against a customized UNITE database [36] with UCLUST, but at a 75% similarity level to remove non-fungal sequences. Any OTU that failed in the third clustering round were removed from the *de novo* OTU list and blasted against another custom database that includes fungal voucher sequences and the UNITE database [14]. OTUs that hit the custom database and passed a quality filter (match length > 0.85, similarity level > 0.75%, and kingdom ‘Fungi’ identity in the taxonomic string) were added back to the *de novo* OTU list. Mapping the original sequence file to the de novo OTU sequence list generated an OTU table. Finally, the abundances of each OTU found in negative controls were subtracted from all samples prior to rarefaction following Nguyen *et al*. (2014)[14].

### Statistical analysis

All samples were rarefied to an equal sampling depth of 18,778 sequences, and observed fungal OTU richness, Chao1, Simpson as well as Shannon indexes for each sample were calculated using the QIIME alpha_diversity.py command. To assess differences among treatments, a series of one-way ANOVAs were applied on OTU richness with Tukey’s multiple comparisons in the R statistical software (www.r-project.org). Diversity estimates, such as Chao 1 was not used in our analyses due to the potential biases associated with them for next generation sequencing studies [37]; however, we have provided them in the supplementary materials (S1 File.) for general comparison. A Bray-Curtis dissimilarity matrix among samples was calculated, and community distance was visualized in non-metric multidimensional scaling (NMDS) plots using the vegan [38] and ggplot2 [39] packages in R. The vegan package was also used to assess potential community composition differences among treatments by testing with ADONIS. For each site, the physical pooled samples sequenced from the standard 0.25 g soil sample (using a 20 ng DNA template amount for PCR) were also compared to a corresponding pool of the individual plot samples (0.25 g soil and 20 ng DNA template) achieved through computation. For this ‘computational’ pool, the number of each OTU recovered from the four individual plot samples were summed, and the resultant composite pool was rarefied at the 18,778-sequence level. We also compared the average occurrence frequency of OTUs between physical and individual pools. The occurrence frequency of each OTU is defined as the occurrence (sequence number ≥ 1) in either 9 physical pools or 24 individual pools in the form of percentage in both site. The average occurrence frequency was calculated as the average of 20 OTUs around each individual OTU starting from the 10^th^ (i.e., 1^st^ to 20^th^ OTU, 2^nd^ to 21^st^ OTU, …, 100^th^ to 119^th^…). Paired T-test was applied between each pair of the 20-OTUs subset (α = 0.05). All T-tests were adjusted with Bonferroni correction with hypotheses *n* = 20.

## Results and Discussion

### High-throughput sequencing of soil fungal communities

After quality filtering and sequence processing, we obtained 2,958,381 sequences, with a range of 18,778 to 78,509 sequences per sample. Rarefying at the lowest per sample recovery threshold (18,778 sequences), we recovered a total of 2,009 unique fungal OTUs from our 66 soil samples. These OTUs represented five known phyla (S2 Fig). We found a total of 1,273 and 1,436 OTUs in CCR (Cedar Creek Ecosystem Science Reserve) and CFC (Cloquet Forestry Center) soils (33 samples at each site), respectively. On average, individual samples contained ~330 OTUs for CCR and ~390 OTUs for CFC. Due to substantial community turnover among samples at each site, soils at CCR and CFC harbored a very high degree of total fungal richness, with rarefaction curves failing to achieve an asymptote (S3 Fig). These results agree with other high-throughput sequencing studies [11,40–42] that consistently reveal extremely high levels of fungal richness in soils of various ecosystems. In fact, only one study to date has successfully saturated the rarefaction curve for soil fungi, recovering a total of 1,002 OTUs from a single biome type [43], a richness level that is considerably smaller than what we detected at our sites.

### Treatment effects on fungal alpha diversity

**Fig 1:**
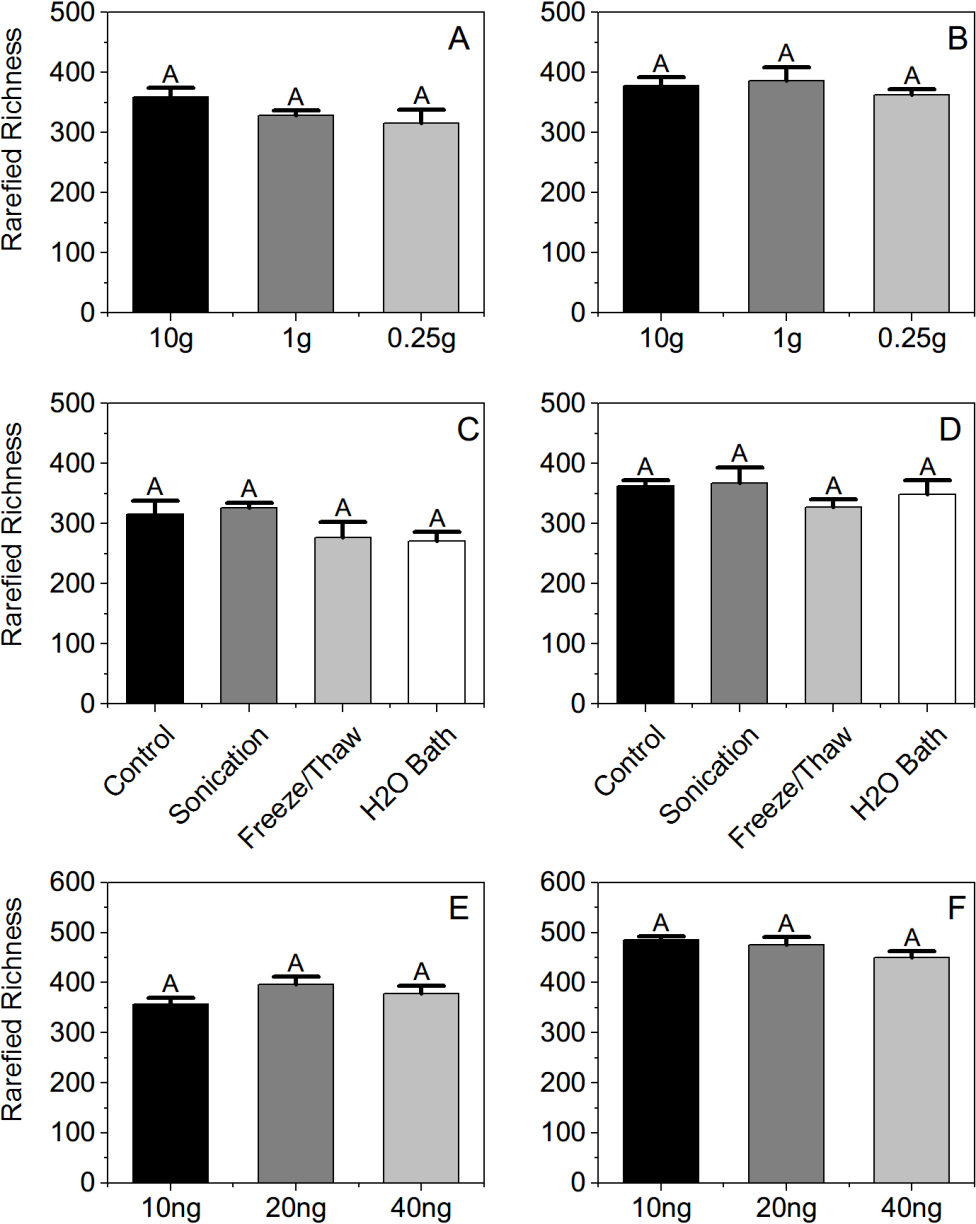
Rarefied OTU richness estimates of soil fungi by treatment. A-B. Amount of soil extracted for CCR and CFC, respectively (n = 5 for each treatment). C-D. Extraction modification for CCR and CFC, respectively (n = 4 for each treatment). E-F. Amount of DNA template in each PCR reactions for CCR and CFC, respectively (n = 3 for each treatment). The same letter above each bar indicates no significant difference (*P* ≤ 0.01) in ANOVA using Tukey’s multiple comparison tests (α = 0.05).

Extracting DNA from larger volumes of soil did not result in significantly higher OTU richness for either site (Fig 1A and 1B for richness and S1 Table for other indexes), although average richness did increase slightly with more soil used in the extraction. Extracting DNA from more soil may be desirable if detecting low abundance taxa is critical. For example, methods for early detection of soil-borne plant pathogens have been developed that require using large volumes of soil [17–19]. In support of this approach, we were able to detect potential plant pathogens, such as *Cochliobolus sativus*, in our 10 g and 1 g soil extraction samples that were not present when only 0.25 g of soil were used. However, our data suggest that extracting DNA from small soil volumes (e.g., the standard 0.25 g recommended in widely-used commercial extraction kits) is sufficient for most HTS applications in which a general characterization of soil fungal richness is required.

Including additional steps in the extraction protocol, such as sonication treatments, freeze/thaw regimes, or period of incubation in a hot water bath, all of which are thought to increase DNA yields and recovered richness, likewise did not significantly impact observed fungal richness (Fig 1C and 1D for richness & S1 Table for other diversity indexes). Although previous studies have found significant effects of DNA extraction method on recovered fungal and bacterial richness [21,24–26,44–46], those studies used older molecular techniques (e.g., RFLP and DGGE) that are likely more sensitive to biases due to the limited depth at which they survey the communities [47–49]. While our results suggest unmodified protocols from standard DNA extraction kits is sufficient for general characterizing fungal richness of soil samples in HTS surveys, we reiterate that study aims must be considered. For example, more stringent DNA extraction treatments may be necessary to detect fungi exhibiting resistant structures [50–52]; however, facilitating extraction of DNA from such structures (e.g., resistant spores) is often undesirable as the associated fungal taxa may represent transient, rather than living, components of the community.

As with the other preparation treatments, the amount of DNA template used in PCR reactions (10 ng, 20 ng, or 40 ng) did not significantly influence recovered fungal richness in our samples either (Fig 1E and 1F for richness & S1 Table for other indexes). While significant effects on fungal richness were not detected overall, opposing trends were noted between sites, with slightly lower richness observed for the highest template amount at CCR and slightly higher richness recorded for higher template amounts at CFC. It is possible that these results are related to the fact that increasing DNA template amount can adversely affect PCR by also increasing the concentrations of inhibitors [27,53,54] and Lindahl *et al*. (2013) noted that diluting DNA template for PCR steps in HTS library preparation is often required [12]. While we did not directly measure potential inhibitors in our samples, our results do highlight the often inconsistent results of increasing DNA templates amounts among different sample types as well as the need for template optimization in preparing HTS runs.

### Sample pooling and treatment effects on fungal community composition

**Fig 2:**
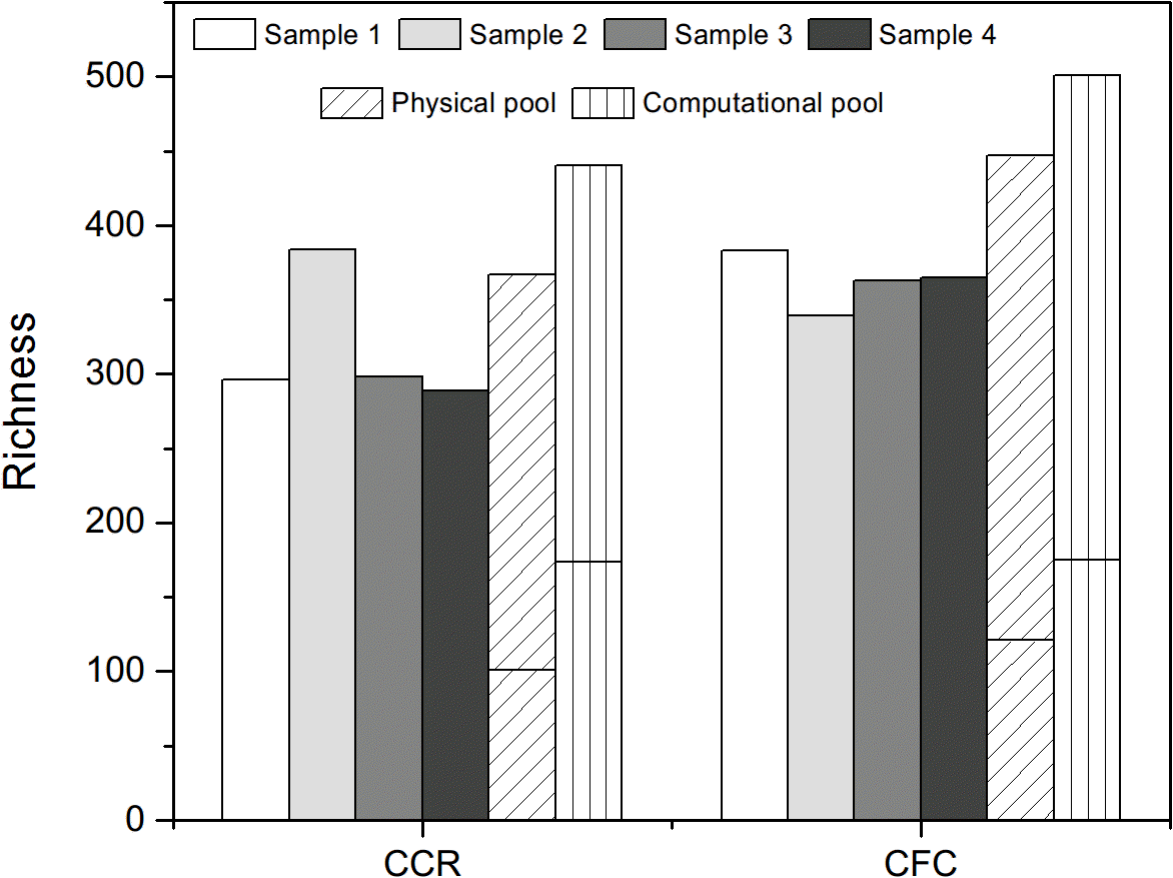
Soil fungal OTU richness of individual samples, physical pool, and computational pool. The shorter bars inside of physical and computational pools indicated the number of unique OTUs that were not shared between the two pools in each site. All samples used 0.25 g soil with standard DNA extraction and PCR procedures.

**Fig 3:**
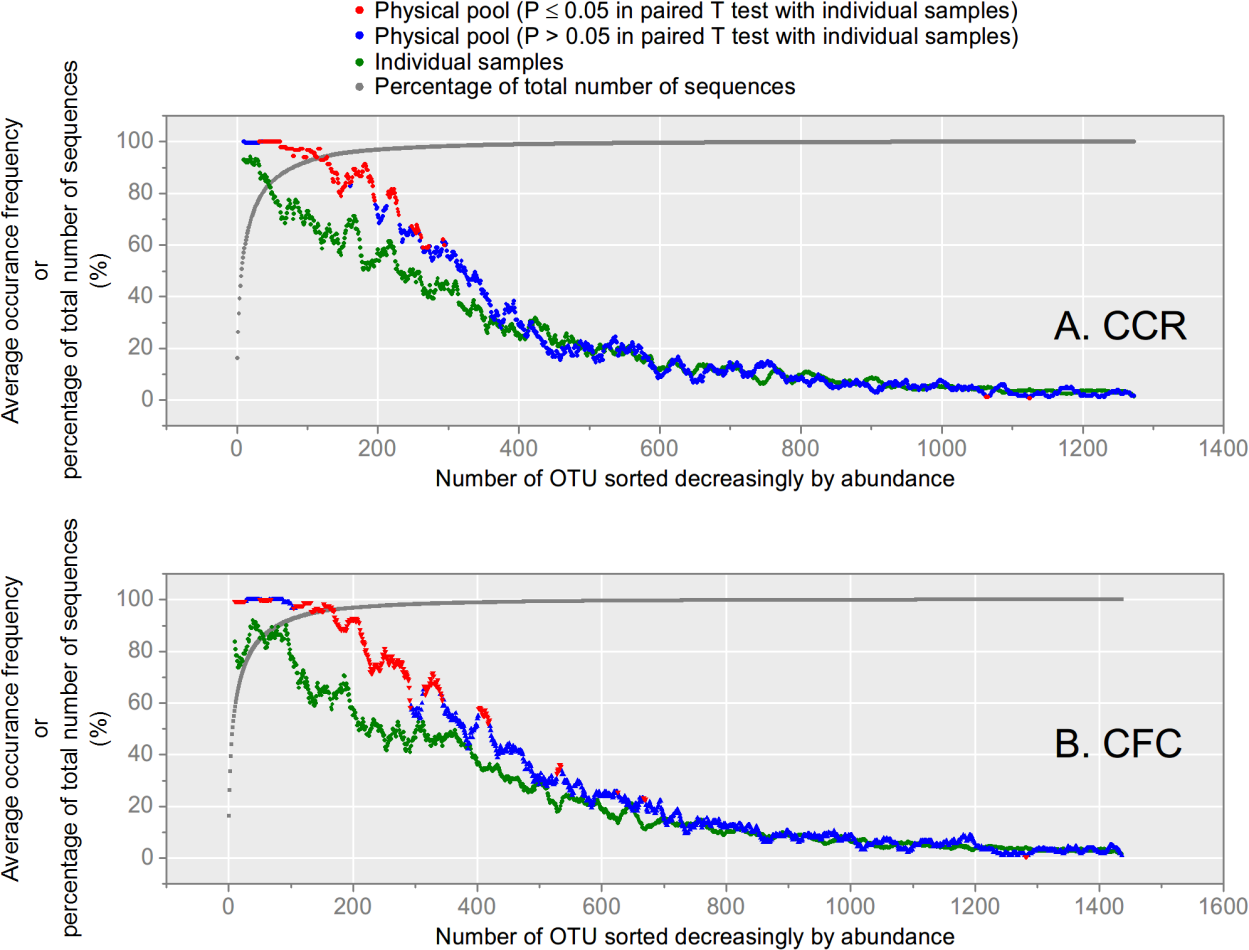
Average occurrence frequency of OTUs in physical pools versus individual samples, and percent of total number of sequence, moving from abundant (left) to rare (right) taxa. Physical pools are in red and blue points, individual samples are in green points, total number of sequence is in gray points. The occurrence frequency of each OTU is defined as the occurrence (sequence number ≥ 1) in either 9 physical pools or 24 individual pools in the form of percentage in both site. The average occurrence frequency shown in the plot was calculated as the average of 20 OTUs around each individual OTU. In the physical pool, red points indicate significant difference (*P* ≤ 0.05) in paired T-test between physical and individual pools (α = 0.05), while blue points were not significantly different. All T-tests were adjusted with Bonferroni correction with hypotheses *n* = 20. The percent of total number of sequence was defined as the sum of abundance percentage from 1^st^ to current OTU in descending order of abundance.

At both sites, physical pools generally had higher richness (Fig 2) and more unique OTUs (S4 Fig) when compared with each individual plot sample. Abundant OTUs (> 1% of the total sequence abundance, consisted of ~70% sequences in total for both sites) appearing in each individual plot sample were also more fully captured in the physical pools at both sites (Fig 3 and S2 File.). Between the two pooling methods, computational pools of each site had higher richness measures than physical pools (Fig 2), due mostly to the contribution of unique OTUs from all four individual plot samples (S4 Fig). A number of OTUs were also recovered in the computation pool that were not present in the physical pool, while a smaller portion were unique to the physical pool alone (Fig 2). Since soil from each of the study sites originated from a very similar source (see methods), it is unlikely that the differences on unique OTUs could be due to data process procedures or PCR amplifications. Our findings are similar to Branco *et al*. (2013), who found that computational pooling yielded higher fungal OTU richness than physical sample pool and Dickie *et al*. (2010), who found different rare taxa in pooled and individual samples [20,55]. Importantly, the high level of fungal community turnover demonstrated in this study and others [11,40–42] indicates the difficultly in achieving comprehensive characterization of soil fungal communities, even using the deep level of survey possible with HTS.

**Fig 4:**
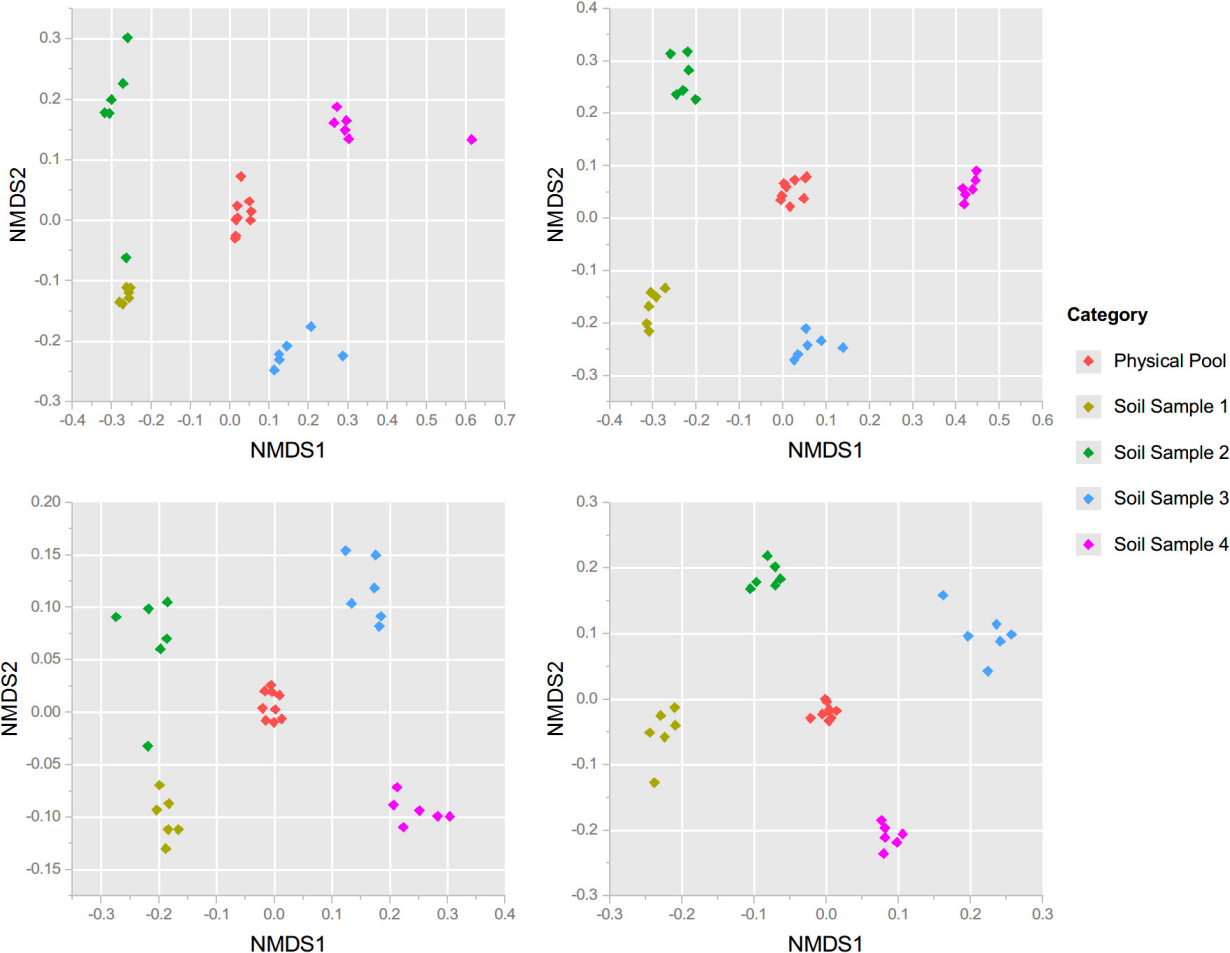
Nonmetric Multidimensional Scaling Plot of physical pools and individual plot samples from each site. Plots based on either Bray-Curtis distance matrices generated from rarefied taxon abundances of A. CCR and B. CFC or from presence/absence matrix of C. CCR and D. CFC.

**Fig 5:**
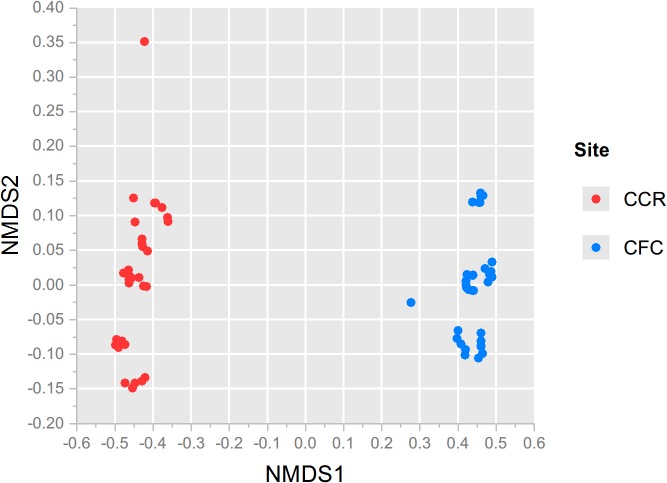
Nonmetric Multidimensional Scaling Plot of samples from CCR and CFC. Plot is based on a Bray-Curtis distance matrix generated from rarefied taxon abundances.

In addition to differences in richness, physically pooled and individual plot samples for each site differed significantly in their fungal community structure (ADONIS based on Bray Curtis and presence/absence distance matrixes respectively, F_4, 28_ = 17.71 and 6.10 for CCR, F_4,28_ = 81.06 and 6.71 for CFC, *P* = 0.001 for all tests). Our results are again similar to those of Branco *et al*. (2013), who showed significant differences in fungal community structure between individual versus physically pooled samples [20]; however, the differences among individual samples taken at their site were not significant. In our study, significant compositional differences among individual samples are apparent in the distinct clustering patterns of the NMDS plots (Fig 4). The importance of individual taxa, rather than their respective abundances, in driving these clustering patterns (Fig 4) is demonstrated by the consistency of the results between the two different matrix types (Bray Curtis vs. presence/absence). In addition, the tight clusters formed by the physical pool samples in the central ‘ordination’ spaces of Fig 4, suggests the averaging effect that physical pooling had on the fungal communities recovered. Overall, site differences in fungal community structure were much stronger than any other effect observed within the sites (Fig 5).

## Conclusions

When considering the effort applied in the sample preparation phase of this survey, we found that the effort generally outweighed any associated rewards. Specifically, we found that sonication, freeze/thaw cycling, and heating during DNA extraction did not result in notable gains of richness or structural characterization of soil fungal communities. Likewise, community-level results were very similar regardless of the amount of soil used in the DNA extraction protocol or the amount of DNA template used in the PCR reactions. We did, however, find that pooling individual soil samples, either physically or computationally, resulted in higher richness. Although computational pooling of samples provides a more comprehensive assessment of local OTU richness than physical pooling, there are additional associated effort of preparation and sequencing. We therefore suggest that for complete biotic inventories, collecting a high number of individual samples at a given site would be preferable, while a single pooled sample from many sites would be preferable for studies aimed at larger spatial scales. Overall, we conclude that following standard protocols of widely used DNA extraction kits is sufficient to successfully assess soil fungal community richness and composition in HTS surveys.

## Supporting Information

S1 Fig Number of publications related to fungal HTS studies from 2000 to 2013 found by searching key words—fungi OR fungal, "high throughput sequencing"—in Google Scholar (http://scholar.google.com/). The points plotted fit an exponential curve (α = 0.05; *P* ≤ 0.01).
(TIF)

S2 Fig Relative abundance of fungal phyla in Cedar Creek Reserve (CCR) and Cloquet Forest Center (CFC) soils after rarefaction (n = 619,674 sequences/site). (TIF)

S3 Fig Taxon (OTU) accumulation curves in this study. Taxon (OTU) accumulation curves depicting the rarefied richness* of fungal taxa as a function of increased sample sequencing effort at CCR (brown) and CFC (green) in the Midwestern United States (*sample depth = 18,778 sequences per sample).
(TIF)

S4 Fig Number of unique and shared OTUs between individual samples or computational pool compared with the physical pool. Samples using 0.25 g soil with standard extraction and PCR procedure were used. Computational pool was generated with the four individual samples and rarefied at the same level of richness (18,778 sequences).
(TIF)

S1 Table One-way ANOVA of alpha diversity indexes and ADONIS in the three treatments on sequencing sample preparations. (DOCX)

S1 File Diversity indexes in different treatments. (XLSX)

S2 File OTU tables for CCR and CFC. (XLSX)
