# Supplementary figures and images for: Effort versus Reward: Preparing Samples for Fungal Community Characterization in High-Throughput Sequencing Surveys of Soils
Source: PLoS One. 2015 May 14;10(5):e0127234. doi: 10.1371/journal.pone.0127234 (PMC4431839; doi:10.1371/journal.pone.0127234)

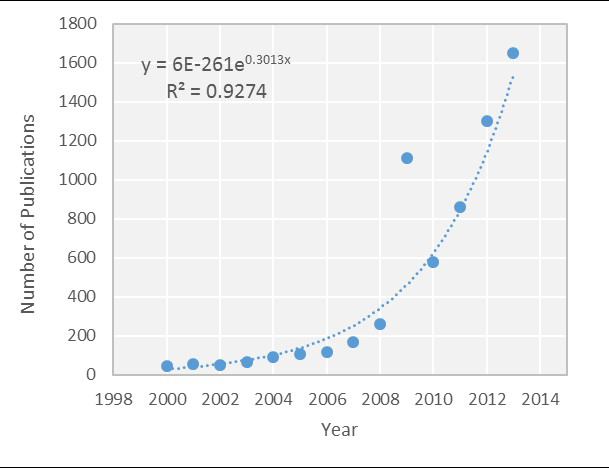

Supplement: S1 Fig — The points plotted fit an exponential curve (α = 0.05; P ≤ 0.01). (TIF) [file pone.0127234.s001.tif]

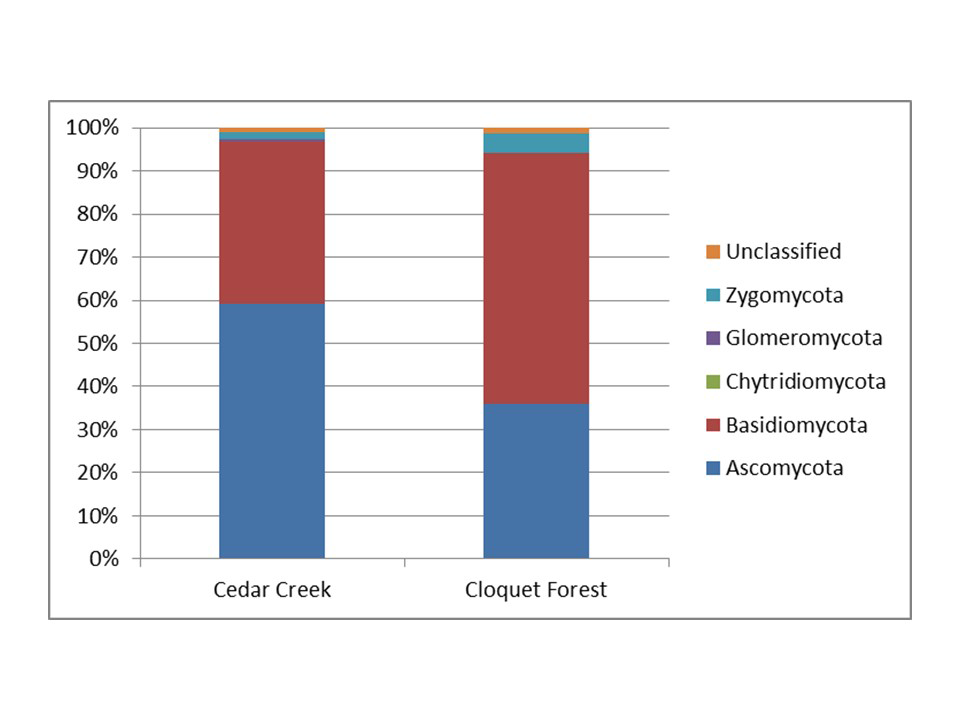

Supplement: S2 Fig — (TIF) [file pone.0127234.s002.tif]

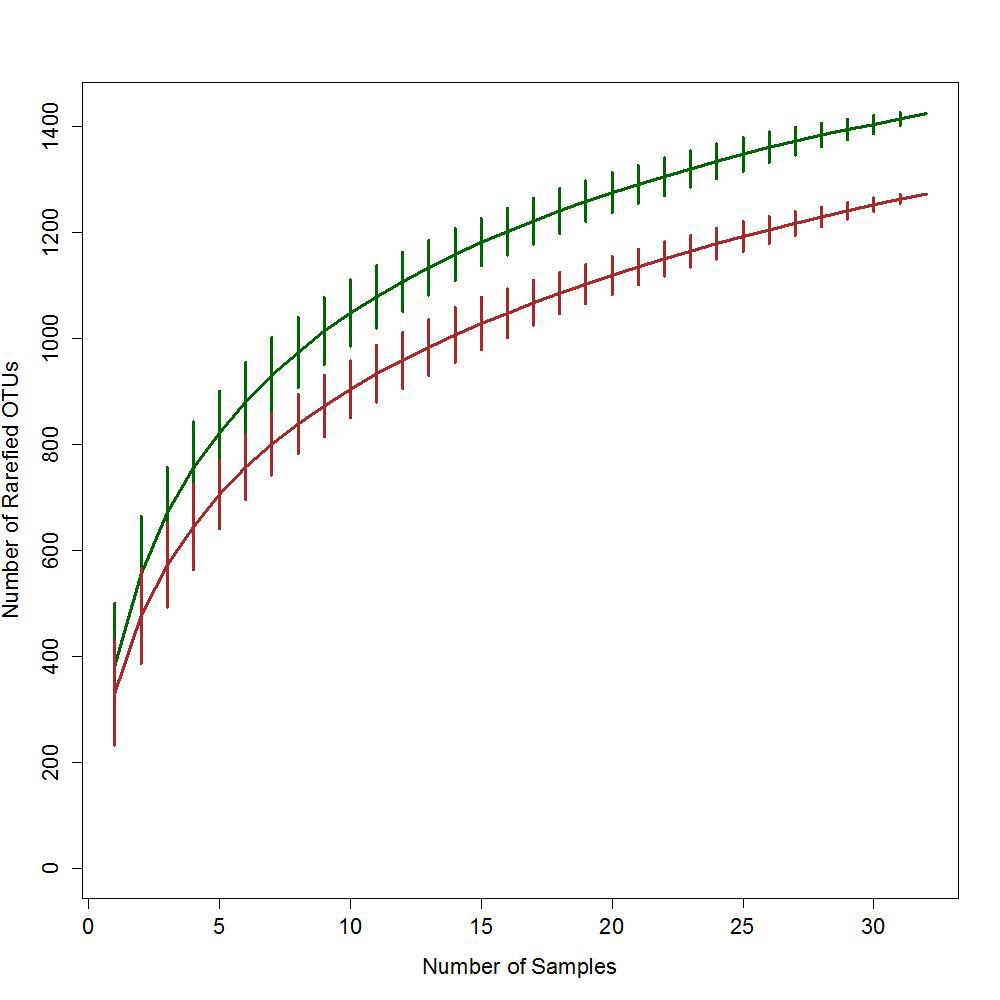

Supplement: S3 Fig — Taxon (OTU) accumulation curves depicting the rarefied richness* of fungal taxa as a function of increased sample sequencing effort at CCR (brown) and CFC (green) in the Midwestern United States (*sample depth = 18,778 sequences per sample). (TIF) [file pone.0127234.s003.tif]
