# Supplementary material for: Effort versus Reward: Preparing Samples for Fungal Community Characterization in High-Throughput Sequencing Surveys of Soils
Source: PLoS One. 2015 May 14;10(5):e0127234. doi: 10.1371/journal.pone.0127234 (PMC4431839; doi:10.1371/journal.pone.0127234)
Supplement: S1 Table — (DOCX) [file pone.0127234.s005.docx]

| Table S1. One-way ANOVA of alpha diversity indexes and ADONIS in the three treatments on sequencing sample preparations. | | | | |
| --- | --- | --- | --- | --- |
| Index | Cedar Creek Reserve (CCR) | | Cloquet Forest Center (CFC) | |
|  | F | *P* | F | *P* |
| *Template amount*  *(n = 3, treatment = 3)* | | | | |
| Richness | 1.77 | 0.249 | 2.49 | 0.164 |
| Simpson | 1.11 | 0.388 | 0.67 | 0.548 |
| Shannon | 0.75 | 0.512 | 0.42 | 0.675 |
| Chao1 | 1.56 | 0.284 | 2.27 | 0.184 |
| ADONIS | 0.07 | 0.987 | 0.29 | 0.918 |
| *Extracting amount*  *(n = 4, treatment = 3)* | | | | |
| Richness | 1.82 | 0.217 | 0.54 | 0.600 |
| Simpson | 1.45 | 0.285 | 0.08 | 0.924 |
| Shannon | 0.90 | 0.440 | 0.003 | 0.997 |
| Chao1 | 1.41 | 0.294 | 1.18 | 0.352 |
| ADONIS | 0.36 | 0.858 | 0.07 | 0.984 |
| *Extraction methods*  *(n = 4, treatment = 4)* | | | | |
| Richness | 2.09 | 0.155 | 0.91 | 0.467 |
| Simpson | 0.31 | 0.820 | 0.36 | 0.786 |
| Shannon | 0.22 | 0.883 | 0.51 | 0.681 |
| Chao1 | 3.22 | 0.062 | 1.00 | 0.426 |
| ADONIS | 0.30 | 0.959 | 1.40 | 0.268 |
|  | | | | |
